# Facial Mimicry and Emotion Consistency: Influences of Memory and Context

**DOI:** 10.1371/journal.pone.0145731

**Published:** 2015-12-23

**Authors:** Alexander J. Kirkham, Amy E. Hayes, Ralph Pawling, Steven P. Tipper

**Affiliations:** 1 Department of Psychology, University of York, York, YO10 5DD, United Kingdom; 2 School of Sport, Health and Exercise Sciences, Bangor University, Bangor, Gwynedd, LL57 2PZ, United Kingdom; 3 School of Natural Sciences and Psychology, Liverpool John Moores University, Tom Reilly Building, Liverpool, L3 3AF, United Kingdom; University of Bologna, ITALY

## Abstract

This study investigates whether mimicry of facial emotions is a stable response or can instead be modulated and influenced by memory of the context in which the emotion was initially observed, and therefore the meaning of the expression. The study manipulated emotion consistency implicitly, where a face expressing smiles or frowns was irrelevant and to be ignored while participants categorised target scenes. Some face identities always expressed emotions consistent with the scene (e.g., smiling with a positive scene), whilst others were always inconsistent (e.g., frowning with a positive scene). During this implicit learning of face identity and emotion consistency there was evidence for encoding of face-scene emotion consistency, with slower RTs, a reduction in trust, and inhibited facial EMG for faces expressing incompatible emotions. However, in a later task where the faces were subsequently viewed expressing emotions with no additional context, there was no evidence for retrieval of prior emotion consistency, as mimicry of emotion was similar for consistent and inconsistent individuals. We conclude that facial mimicry can be influenced by current emotion context, but there is little evidence of learning, as subsequent mimicry of emotionally consistent and inconsistent faces is similar.

## Introduction

Adapting and integrating to our current environment through physical and social imitation of those around us often seems to be an unconscious process where “one typically does not notice doing these things–if at all–until after the fact” [1]. The swift adaptation of our own facial expressions to mimic the emotional expressions of others may be of most importance for facilitating rapid social cohesion through improved bonds [2] and liking [3]. The current study further investigates the processes mediating mimicry of emotions such as smiles and frowns. It first examines whether implicit contextual cues, where an individual’s expressed emotion consistently matches or mismatches the emotional status of the environment, influence mimicry and judgments of a person’s trustworthiness. Second, it asks whether the incidental learning of whether a person’s emotions are always appropriate or always inappropriate to the emotional properties of the environment can be retrieved at a later time and influence whether mimicry takes place or is suppressed.

### Mimicry: non-conscious and adaptive function?

It can be posited that emotional mimicry may be based upon an automatic and intrinsic process, strongly linked to automatic processing of nonverbal communications through expressions [4]. Indeed, a body of research has argued that emotional mimicry often occurs rapidly (within 500ms of expression exposure–[5–6]) and without substantial consideration, being “nonconscious, unintentional, and effortless” [7]. It can still be observed when an emotional expression is presented subconsciously [8–10], whilst the expression is irrelevant to the main task [11], and whilst being told to suppress or inhibit any facial movement [6, 12].

However, if emotional mimicry was purely automatic this may result in inappropriate responses in certain social situations. For example, if a person is smiling for what we consider to be negative reasons we *should not* mimic that smile, or indeed if we have previously encountered a person with whom we do not share similar emotional responses, emotional mimicry may not be appropriate. This therefore leads to a further viewpoint that emotional mimicry is not truly automatic, nor entirely self-guided, but instead may be a form of moderated automaticity, or a combination of automaticity and “controlability” [4]. Indeed, it has been proposed that emotional mimicry may only occur when there is a neutral or positive relationship between the parties [13], when the mimicker has a positive attitude toward the expresser [14], when sharing the same group membership [15], or when cooperation rather than competition is expected with the other party [16]. Furthermore, other contextual factors have been found to manipulate the extent to which mimicry is shown toward emotional expressers, such as the current mood and emotions felt by participants [17], perceived fairness of the emotion expresser [18], and task relevance [11].

### The meaning of the expression and learning about the expresser

The majority of research argues that emotional mimicry is *either* automatic or a form of moderated-automaticity influenced by contextual factors, and there appears to be minimal research conducted into how emotional mimicry may be influenced by the *interpretation and meaning* of the viewed emotion. A notable exception by Halberstadt and colleagues [19] shows how emotional expressions when viewing ambiguous faces (composed from a morph of smiling and anger stills) are determined by associating the ambiguous face with terms such as “happy”. More recent work has also demonstrated that mere association of a neutral face with an emotional term can result in mimicry of that emotion, even though the face shows no indication of this [20]. Traditionally however, and certainly in terms of these aforementioned studies, smiles are indicative of positive emotions, and frowns of negative emotions, therefore resulting in a *consistent* mimicry response. However, there is relatively little research concerning *inconsistent* emotional signals, for example where a smile is positive for the expresser yet negative to the viewer because it is inappropriate in the current context.

A further issue concerns the *learning* of emotional response consistencies. We learn about the world in two ways, either implicitly where we are *not* told about particular properties of the world but detect them even when the critical stimulus is irrelevant to our current goals. Or we learn explicitly, where another individual can direct our attention to particular properties of the environment and there is conscious reportable learning (see [21], for review).

In the present study we examine implicit learning and subsequent memory encoding through a target categorisation task. Faces expressing positive and negative emotions are presented alongside positive and negative scenes; participants are to only categorise the scenes as being positive or negative as quickly as possible, while they are informed that the faces are irrelevant and to be ignored. Half of the face identities always express consistent emotions, smiling at positive scenes and frowning at negative scenes, whereas the remaining individuals always express inconsistent emotions that mismatch the to-be-classified scene. Most importantly, participants are told the faces are irrelevant to their task, can be ignored, and they are never informed about the relationship between the face and scene emotion. Note that this is implicit in the sense that facial identity and expression are *irrelevant to the participant’s goals*; we are not necessarily making the further claim of unconscious learning.

In a subsequent task only the faces are shown and are to be categorised as smiling or frowning; is mimicry of emotion influenced by memory of the prior emotional consistency of particular face identities? If implicit learning has taken place when the emotional consistency of a particular face is encoded, even while ignoring the face, then we predict significant reductions in the mimicry of such an unreliable and inconsistent person. In this sense we predict that the meaning of the target expression, as previously implicitly learnt according to the emotional consistency of each face, will be retrieved from memory and influence how much emotional mimicry is evoked.

As noted, there are two main stages to this study. The first stage involves classifying target scenes as being positive or negative. Whilst doing so, implicit learning of the emotional response consistency of a concurrently presented irrelevant face takes place. The second stage involves classifying the expressed emotion of a target face with no additional contextual information. During this second exposure, the retrieval of the face identity and previous emotional consistency should be drawn from memory, and is anticipated to influence any mimicry of the face emotion displayed. That is, memory of faces that previously produced inappropriate emotions may suppress the amount of mimicry shown at this later time, whereas memory of faces that produced appropriate emotions may elicit strong mimicry effects.

It is important in such a study to confirm that during implicit learning the relationship between the face emotion and target scene emotion is being computed and encoded into memory. Therefore we have three measures of consistent vs. inconsistent facial emotions during learning. Firstly, reaction times (RT) to classify the scene as positive or negative. We predict that when the scene and face emotion are inconsistent there will be response competition. For example, when the scene requires a negative classification response, a smiling face will evoke the opposite response and slow down RT. Second, we take measures of face trustworthiness both before and after learning. We predict that faces expressing emotions inconsistent with the scene will become less trusted. This reduced trust should impair subsequent mimicry during retrieval since emotional mimicry is thought to only occur with a neutral or positive relationship between the parties [13–16]. And third, we predict that when there is a conflict between the facial emotions, such as smiling with a negative scene, this will impair facial mimicry as measured via EMG. If we can detect these on-line measures of emotional consistency during learning, this will demonstrate that face emotion consistency was computed and enable tests of whether it is retrieved and influences mimicry at a later point in time.

## Methods

### Subjects

28 undergraduate female students voluntarily participated in the study in return for £6 payment. All subjects had normal or corrected-to-normal vision and were aged between 18 and 22 years of age (mean age of 20.05 years). All participants provided written consent and the research was given ethical approval by the Departmental Ethics Committee of the University of York Psychology Department. All participants were debriefed following completion of the study.

### Stimuli & Presentation

Eight faces (four males and four females) selected from two headshot databases (KDEF–[22]; and NimStim–[23]) were presented to participants. All faces were seen as a static image with a neutral expression or as morph sequences from neutral to smiling expressions and from neutral to frowning expressions. Each morph sequence lasted 300ms and comprised of 12 individual images displayed for 25ms each to create a naturally timed expression change.

A further ten images for use in the task were taken from the IAPS database [24]. All selected images (five positive scenes [nos. 1460, 5199, 5764, 5825, and 5833] and five negative scenes [nos. 1271, 9000, 9471, 9495, and 9600]) were moderately positive or negative, and avoided extreme emotion samples. Negative images included scenes such as a sinking ship and burnt buildings, whilst positive images included scenes such as a countryside landscape and a bright ocean beach scene.

All stages of the experiment were presented using E-Prime 2 [25]. Participants were seated approximately 60cm from the monitor. Viewed faces had a visual angle of approximately 13° x 13°, whilst all scenes were approximately 11°x 9°.

Participants were offered short breaks between each stage, and the overall duration of the experiment including setup and debrief took no longer than 1 hour.

### Procedure

**Fig 1:**
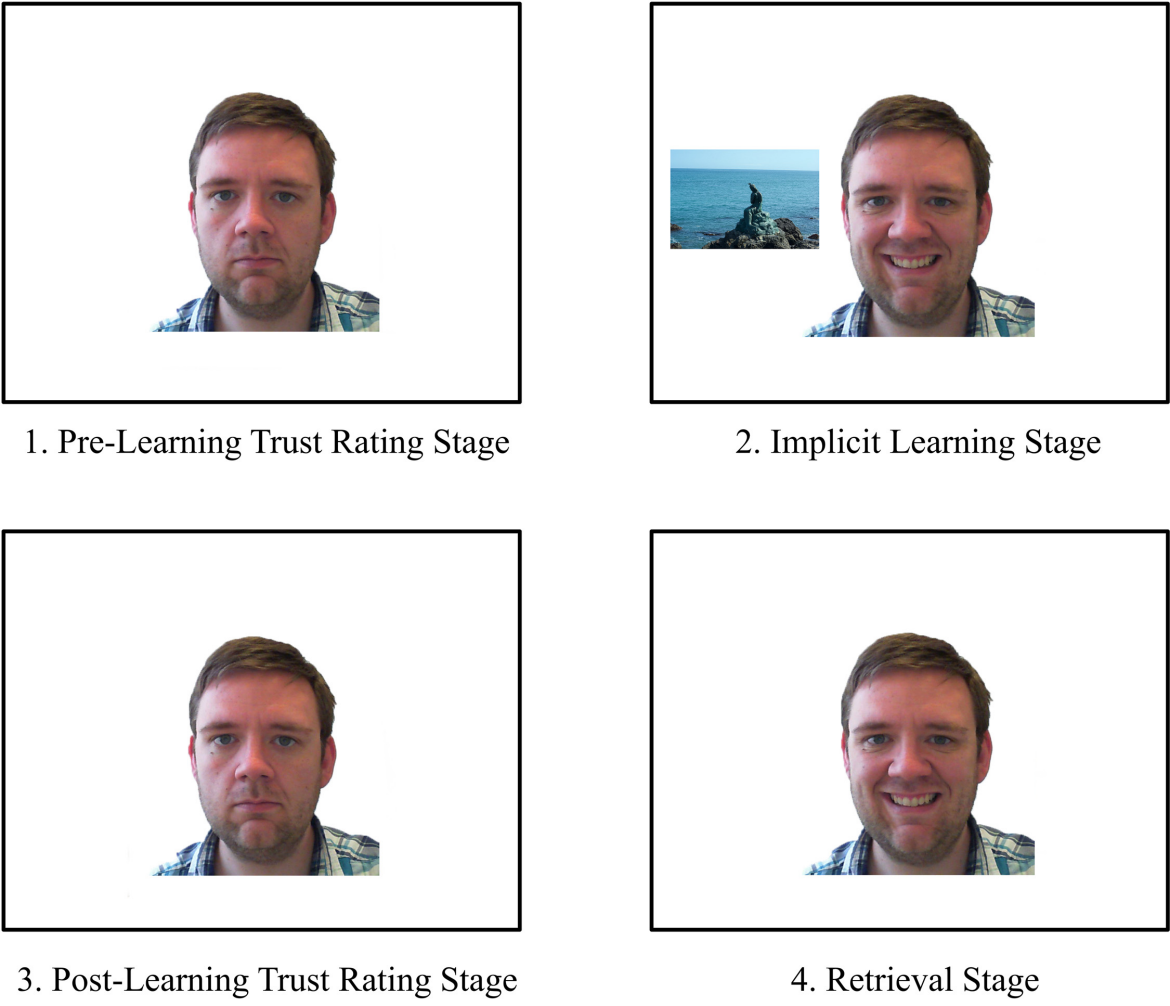
Examples of the four stages of the experiment. The face and target object are not shown to scale, specific details for the stages are provided within each section.

There were four stages to the study, (see Fig 1). Specific methodologies for each stage will be detailed under the applicable headings. The individual shown in the images present in this manuscript has given written informed consent (as outlined in PLOS consent form) to publish these case details.

#### Pre-learning trust rating stage

Participants rated each face according to perceived trustworthiness. Each rating was performed using a mouse-click upon a single horizontal line shown on the screen. The line had no indicated values other than end caps showing—or +. The coordinates of the mouse-click were converted to represent a rating of between -99 and +99.

**Fig 2:**
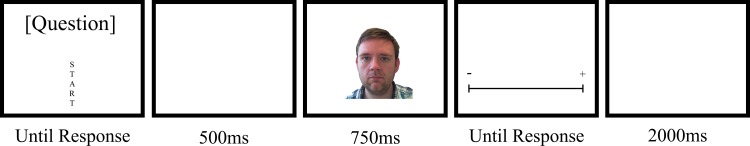
A standard trial during the Rating Stages (1 and 3). All faces were presented centrally with a neutral expression for the timing durations shown. ‘Question’ refers to: “How trustworthy is this person?”

Participants clicked on a start button located centrally on the screen to initiate each trial. This ensured that on all trials participants repositioned the mouse each time, thus reducing the likelihood of uniform ratings. Participants rated each face twice, and all faces were presented centrally with a neutral expression. There was no time limit for the ratings to be provided but participants were encouraged to go with their first reaction. See Fig 2 for an example trial.

#### Implicit learning stage

Participants categorised each IAPS scene as positive or negative. Each scene was shown once in a training block to ensure that all participants were familiar with the scenes, and could correctly determine each as positive or negative. Positive and negative categorisations were made using key-presses (Y for positive and B for negative).

**Fig 3:**
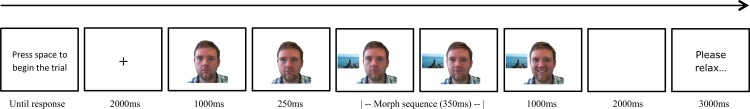
Example of a Learning Stage trial. Note that these scene and face images are not presented correctly to scale, in order to improve clarity; during the study the scene height was approximately 60% of the face height, please refer to the visual angle details in Stimuli and Presentation.

Throughout the main blocks of trials participants were shown each scene alongside a concurrently presented face (see Fig 3). Participants were instructed to ignore the face, since it would distract them from their task goal. All responses required the same key-presses as during the training trials.

Each trial was initiated with a key-press followed by a fixation screen for 2000ms. A neutral-expression face was presented in the centre of the screen for 1000ms. This somewhat long presentation enabled more time for encoding identity to facilitate the learning of identity and emotion appropriateness. As no emotion was expressed during this period it was not included in EMG analysis of emotion mimicry. After this gaze direction shifted toward the left or right for 250ms to provide a gaze-cue to the forthcoming scene location. Following this, the face returned to a central gaze before morphing from the neutral expression to a full expression (either smiling or frowning). The expression morph duration was 350ms (including the aforementioned central gaze neutral expression which was displayed for 50ms). The scene was presented 100ms into the morph sequence and remained displayed alongside the face. After the morph was fully completed, the face remained on-screen (with full expression) for a further 1000ms. A blank screen was then presented for 2000ms. The participant could respond (categorising the scene as positive or negative) at any time from when the scene first appeared, through until the blank screen had elapsed. If they responded incorrectly (or did not respond) an error tone was heard at the end of the blank screen from speakers positioned behind the monitor. Finally, a screen instructing the participant to relax was presented for 3000ms before the next trial began (see Fig 3 for example).

During this stage each individual face exhibited *either* a consistent or inconsistent expression. Consistent expression faces always smiled with positive scenes and frowned with negative scenes; inconsistent expression faces always smiled with negative scenes and frowned with positive scenes.

The eight faces (four males and four females), were divided in to four consistent expression faces (two males and two females) and four inconsistent expression faces. The face expression-consistency assignment was counterbalanced across all participants. Each face was presented alongside each scene, making a total of 80 trials. For later purposes these were split across five bins each containing 16 trials (with each face presented twice in a block).

#### Post-learning trust rating stage

Participants again rated the faces for trustworthiness using the same function as detailed in the Pre-learning trust rating stage. This second rating of the faces was to determine if implicit learning of personality and emotion consistency characteristics had been achieved, in that these factors had been committed to memory.

#### Retrieval stage

**Fig 4:**
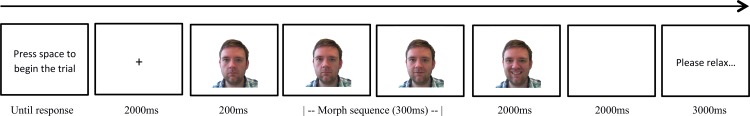
Retrieval Stage trial. A typical trial from the Retrieval Stage with details of timings.

On each trial participants categorised each of the previously seen faces as exhibiting either a smile or a frown (morphing from a neutral expression to the target expression) using a key-press response. Each trial was initiated with a key-press before being presented with a fixation screen for 2000ms. A neutral-expression face was presented in the centre of the screen for 200ms before morphing from the neutral expression to a full expression (either smiling or frowning). The expression morph duration was 300ms. After the morph was fully completed, the face remained on-screen (with full expression) for 2000ms. Following this a blank screen was presented for 2000ms. The participant could respond (categorising a smile or frown) at any time from when the face first appeared, through until the blank screen had elapsed. If they responded incorrectly (or did not respond) an error tone was heard at the end of the blank screen. Finally, a screen instructing the participant to relax was presented for 3000ms before the next trial began (see Fig 4 for details).

Participants viewed each face a total of eight times (four smiling, and four frowning), resulting in a total of 64 trials. Although we refer to this as the retrieval stage, the participants were not aware of any retrieval element to the task. As such it refers to the retrieval from memory of their past experience with each face, and may highlight mimicry differences between faces that previously showed consistent and inconsistent expressions through the EMG format. Note that this stage includes no contextual information, nor indication of each face’s previous emotional consistency.

### EMG Apparatus and Methodology

The dominant method of assessing facial mimicry is through the use of electromyography (EMG). This method has the benefits that it can be non-invasive and is extremely sensitive, thereby being able to detect responses under the visual detection threshold [26]. The specific muscles responsible for portraying different expression states are also well researched, with the zygomaticus major responsible for smiles through cheek movement, and the corrugator supercilii responsible for frowns through brow movement [27]. The binding of corrugator activity with negative emotions, and zygomaticus activity with positive emotions are a well-researched outcome [28].

Facial electromyographic (EMG) activity was measured from the zygomaticus major and corrugator supercilii muscles at a resolution of 2000Hz using a Biopac system comprising of an MP150 controller and two EMG100C MRI modules. Two pairs of 4mm Ag/AgCl electrodes filled with conductive electrolyte gel were secured upon the left-hand side of the face of each participant using adhesive discs. Electrodes were sited to record activity from the zygomaticus major and corrugator supercilii, with an additional ground electrode placed upon the forehead [26].

Following the completion of each recording, the raw signal from each muscle was filtered using a bandpass filter (20Hz - 500Hz) and a notch filter of 50Hz, before being rectified and smoothed with an integration window of 50ms.

All EMG analyses were performed upon each muscle separately, since there are often substantial differences in the reactivity of the zygomaticus (cheek) and corrugator (brow) muscles due to the differences in overall muscle size and inherent differences in automaticity of reaction [29–30]. EMG activity was measured across each trial as a percentage ratio between mean muscle activity during the final 500ms of the fixation screen (to be treated as a baseline), and subsequent 500ms time windows. EMG activity was recorded for 4000ms following the fixation period of each trial. Within the implicit learning stage the core focus is to detect mimicry when viewing a face expressing emotion, therefore the first 1000ms where the face did not express any emotion, was not analysed. Thus six individual windows of 500ms each were analysed. In the retrieval stage eight individual windows of 500ms each were analysed, as this stage did not have a 1000ms neutral expression period.

### Trial and Participant Rejections

Due to the sensitivity of EMG recordings it was necessary to remove some trials, and hence participants from the EMG analyses. Trials were rejected where it was evident that substantial and unrelated movements had occurred (e.g. sneezing or coughing); this was performed by the researcher who was blind to the trial condition. Such instances were identified by large-scale anomalous fluctuations in the EMG time-course, or muscle activity that was substantially greater during the fixation period. Clearly such instances do not affect behavioural data; yet the effect on EMG data can be dramatic. Therefore whilst EMG data in these instances has been removed, there is no reason to similarly reduce the behavioural data. Data from three participants was removed as these contained an excessive number of movement-rejected trials. Data from one further participant was removed because they had previously performed a similar study. Participants were naïve as to the true purpose of the EMG apparatus, and therefore the unrelated movements detailed are unavoidable as participants can on occasion make no effort to minimise these.

## Results and Discussion

### Pre/Post-Learning Trustworthiness

**Fig 5:**
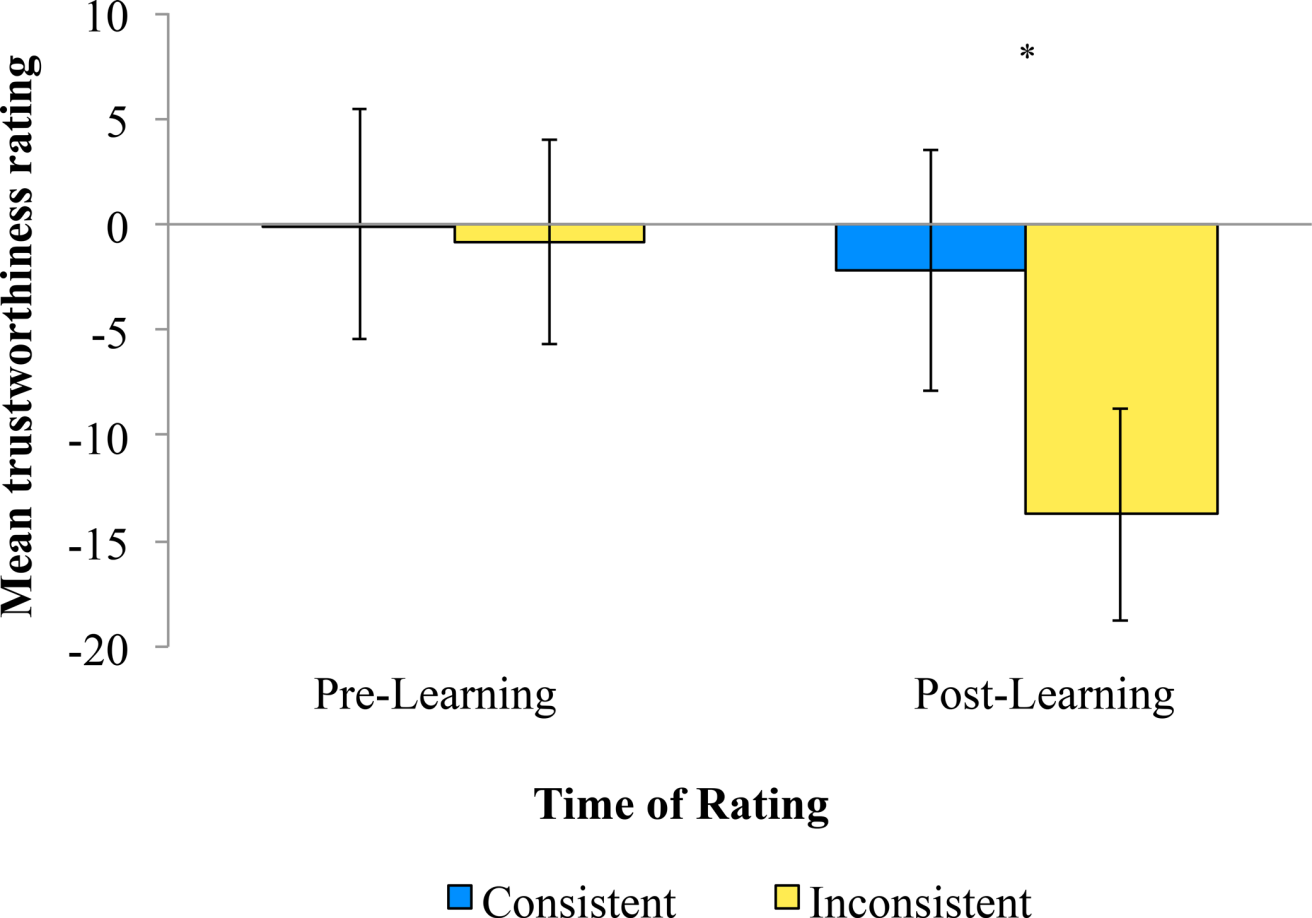
Trustworthiness ratings. Face trustworthiness both before and after the learning stage had been completed. Error bars denote SE.

All 28 participants rated each face for trustworthiness, both before and after the implicit learning-stage. Rating measures were analysed using a 2 x 2 repeated measures ANOVA, with factors of Consistency (Consistent/Inconsistent emotion) and Time (Pre/Post-learning stage). As predicted, there was a tendency for faces with Consistent expressions (M = 1.09, SE = 4.88) to be rated as more trustworthy than those with Inconsistent expressions (M = -7.29, SE = 4.35) [*F*(1,27) = 3.64, *p* = .067, η_p_^2^ = .12]. Of most importance, there was a significant interaction between the Consistency of emotional response and the time of rating [*F*(1,27) = 5.42, *p* = .028, η_p_^2^ = .17] (see Fig 5).

Examining this result further, during the pre-learning stage rating Consistent (M = -0.01, SE = 5.42) and Inconsistent (M = -0.84, SE = 4.83) faces were found to have highly similar trustworthiness ratings [*t*(27) = 0.24, *p* = .81]. However, during the post- learning stage rating faces that expressed emotions that were Inconsistent (M = -13.75, SE = 5.01) with the valence of the target scene were rated as less trustworthy than those with Consistent expressions (M = -2.17, SE = 5.74) [*t*(27) = 2.59, *p* = .015].

### Implicit learning Stage

All error trials and those with RTs of < 200ms or >2500ms were removed prior to any further behavioural analysis to remove any responses that were too quick for reasonable perception and too slow to indicate full concentration. For clarity all further analysis is as follows: Consistent trial = a positive scene with a smiling face / a negative scene with a frowning face; Inconsistent trial = a positive scene with a frowning face / a negative scene with a smiling face. Due to a corrupt file, data from 1 participant is not available, leaving data from 27 participants available for analysis.

#### Reaction times

**Fig 6:**
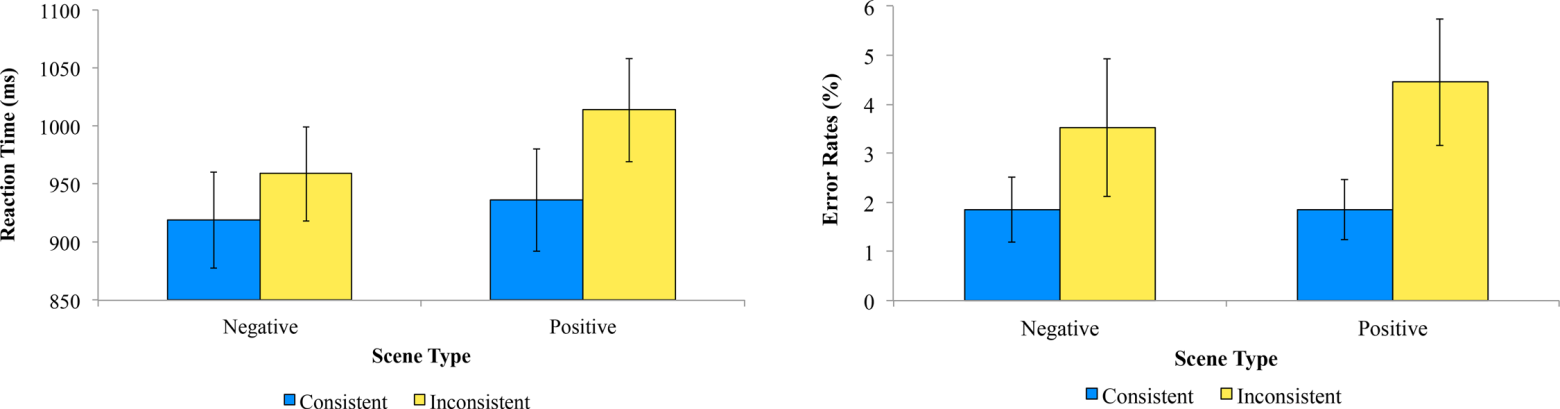
Learning Stage. Graphs illustrating the reaction times and error rates toward scene and face expression-consistency pairings. Error bars denote SE.

A 2 x 2 repeated measures ANOVA was performed on the data (Consistency: Consistent / Inconsistent; Scene: Positive / Negative). Consistent trials had faster RTs (M = 928ms, SE = 41.79) compared to Inconsistent trials (M = 986ms, SE = 41.26) [*F*(1,26) = 21.71, *p* < .001, η_p_^2^ = .46], whilst negative scenes had faster RTs (M = 939ms, SE = 40.21) compared to positive scenes (M = 975ms, SE = 42.80) [*F*(1,26) = 8.21, *p* = .008, η_p_^2^ = .24]. There was no interaction between Consistency and Scene [*F*(1,26) = 1.56, *p* = .22, η_p_^2^ = .057], indicating that the RT differences between the Scenes (moderately positive / negative) were stable across each Consistency form (see Fig 6). This RT emotion compatibility effect is reminiscent of that observed by [31].

#### Accuracy

Analyses were identical to those conducted with the RT data. Inconsistent trials (M = 3.98%, SE = 1.22) resulted in marginally more errors than consistent trials (M = 1.85%, SE = 0.51) [*F*(1,26) = 3.71, *p* = .065, η_p_^2^ = .12]. There was no main effect of Scene (positive: M = 3.15%, SE = 0.74; negative: M = 2.69%, SE = 0.91) [*F*(1,26) = 0.46, *p* = .50, η_p_^2^ = .017], nor an interaction of Consistency and Scene [*F*(1,26) = 0.46, *p* = .50, η_p_^2^ = .017] (see Fig 6).

#### EMG data

Although this stage of the study was designed to build up representations and judgements of each identity, based upon their expression consistency, it also has a secondary function. We already know that emotional mimicry can occur even when the participant is performing a task unrelated to a facial expression [11]; therefore participants may also demonstrate mimicry of any observed expression if automatic mimicry is occurring. However, any mimicry may also be dependent upon the contextual factors of the task; for example, mimicry of a smiling face might be more likely in the context of a positive image, whereas such mimicry of a smiling face might be suppressed in the presence of a mismatching negative scene.

Analyses were conducted using a 3-way repeated measure ANOVA with factors of Expression (smile and frown), Consistency (consistent and inconsistent), and Time (6 time periods).

#### Corrugator

There was no main effect of Expression [*F*(1,23) = 2.26, *p* = .15, η_p_^2^ = .089], or main effect of Consistency [*F*(1,23) = 2.41, *p* = .13, η_p_^2^ = .095]. A main effect of Time was observed [*F*(5,115) = 7.77, *p* = .001, η_p_^2^ = .25]. Of most importance, there were significant interactions of Expression and Consistency [*F*(1,23) = 5.20, *p* = .032, η_p_^2^ = .18], and also Expression, Consistency and Time [*F*(5,115) = 4.15, *p* = .012, η_p_^2^ = .15]. To analyse the interactions further, separate analyses of consistent and inconsistent emotions were undertaken.

**Fig 7:**
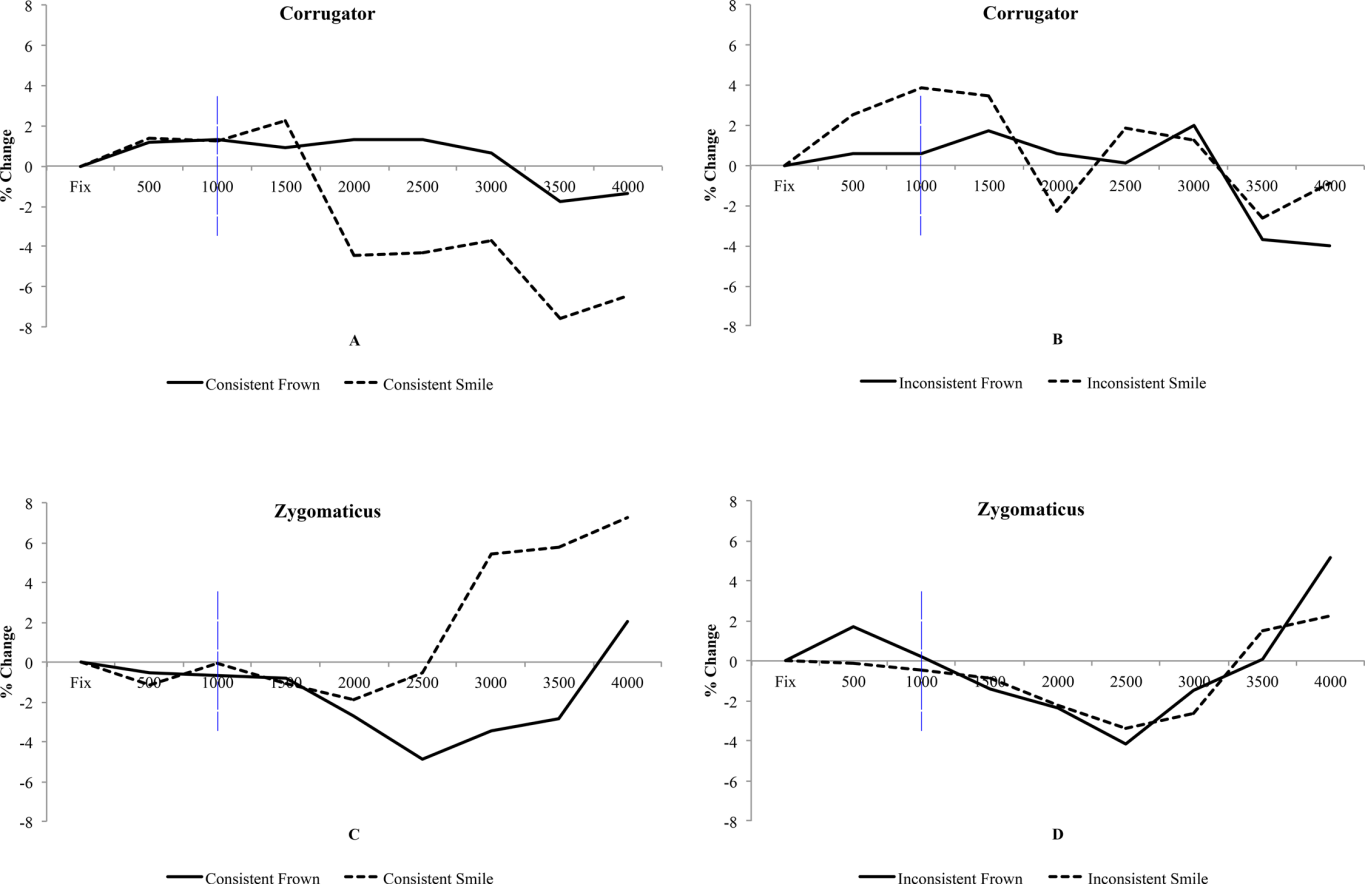
Implicit Learning Stage EMG. Time course graphs detailing muscle activity from the corrugator (A and B) and zygomaticus (C and D) toward Expressions and factors of Consistency. Blue vertical line illustrates the start point of data analysis to detect emotion mimicry.

A repeated measures ANOVA was conducted on the *consistent* expressions data, with 2 levels of Expression and Time as contributing factors. Viewing frowning faces produced relatively greater activity in the corrugator muscle compared to viewing smiles as emotion mimicry predicts [*F*(1,23) = 5.55, *p* = .027, η_p_^2^ = .19]. A main effect of Time [*F*(5,115) = 7.45, *p* = .001, η_p_^2^ = .25], and an interaction of Expression and Time were also observed [*F*(5,115) = 8.34, *p* < .001, η_p_^2^ = .27]. Viewing frowns produced greater relative activity compared to smiles (*p* < .05) in the 2000-4000ms time periods as revealed by paired t-tests (see Fig 7A), and this is the standard effect observed in the literature.

As expected, there was no significant difference between the *inconsistent* frowns and inconsistent smiles [*F*(1,23) = 0.25, *p* = .62, η_p_^2^ = .011]. A main effect of Time was observed [*F*(5,115) = 6.05, *p* = .003, η_p_^2^ = .21]. Finally, although an interaction of Expression and Time was observed [*F*(5,115) = 3.32, *p* = .020, η_p_^2^ = .13], subsequent planned contrasts only detected a marginal effect in 3500–4000 period (p = .06) where smiles produced more activity than frowns.

During the learning-stage corrugator activity is influenced by the consistency of the face emotion and target stimulus. Thus when emotions are *consistent* the standard mimicry effect is observed where the brow (corrugator) is more active when viewing a frowning face as compared to a smiling face. However, when the face emotion and target mismatch, such as frowning at a pleasant object, there is a tendency for the opposite pattern.

#### Zygomaticus

Analyses were conducted identically to those of the corrugator. As expected, viewing smiles evoked more zygomaticus activity than when viewing frowns, with a marginal effect in the predicted direction [*F*(1,23) = 4.15, *p* = .053, η_p_^2^ = .15], whilst there was no main effect of Consistency [*F*(1,23) = 0.45, *p* = .51, η_p_^2^ = .019]. A main effect of Time was also observed [*F*(5,115) = 5.04, *p* = .015, η_p_^2^ = .18]. Although there was no significant interaction of Expression and Consistency [*F*(1,23) = 2.77, *p* = .11, η_p_^2^ = .11], this interaction was significant over Time [*F*(5,115) = 3.17, *p* = .049, η_p_^2^ = .12].

To explore the main issue of emotional consistency further, analyses were conducted individually for each consistency condition. As predicted, *consistent* smiles produced a significant increase in relative activity over *consistent* frowns [*F*(1,23) = 4.38, *p* = .048, η_p_^2^ = .16], whilst a main effect of Time was also obtained [*F*(5,115) = 3.70, *p* = .031, η_p_^2^ = .14]. Expression was found to vary over Time [*F*(5,115) = 3.82, *p* = .019, η_p_^2^ = .14], with activity during the 3000ms and 3500ms time periods producing a significant increase in relative activity to smiles compared to frowns (*p* = .050 & *p* = .010 respectively). Time periods 2500ms and 4000ms produced similar differences and approached significance (*p* < .1) (see Fig 7C).

In contrast, and as expected, within *inconsistent* expressions there was no main effect of Expression [*F*(1,23) = 0.23, *p* = .88, η_p_^2^ = .001]. A main effect of Time was found [*F*(5,115) = 4.99, *p* = .018, η_p_^2^ = .18] but this did not interact with Expression [*F*(5,115) = 0.72, *p* = .47, η_p_^2^ = .030] (see Fig 7D).

In summary, as can be clearly seen in Fig 7, the consistency of expressed emotion influences mimicry. That is, in the traditional analysis comparing smiles versus frowns in each muscle, a significant contrast is detected in both the corrugator and zygomaticus muscles *only* for emotions consistent with the context. In contrast, mimicry is suppressed when viewing a face expressing an emotion that is inappropriate in the current context.

#### Analysis within emotion

The above analysis is the traditional approach that demonstrates that each muscle mimics the viewed emotion. That is, the zygomaticus cheek muscle associated with expressing smiles is more active when viewing smiling faces, whereas the corrugator brow muscle associated with negative emotions is more active when viewing frowns. However, to investigate the effects of emotion consistency we felt it worthwhile to analyse within an emotion. For example, comparing the response to consistent and inconsistent smiles within a muscle.

#### Smiles

Corrugator: Viewing inconsistent smiles produced significantly greater muscle activity compared to consistent smiles [*F*(1,23) = 6.58, *p* = .017, η_p_^2^ = .22]. A main effect of Time was also obtained [*F*(5,115) = 10.02, *p* < .001, η_p_^2^ = .30], which interacted with the consistency of the smile [*F*(5,115) = 3.14, *p* = .038, η_p_^2^ = .12].

Zygomaticus: There was no main effect of consistency for smile expressions [*F*(1,23) = 2.11, *p* = .16, η_p_^2^ = .084]. A main effect of Time was obtained [*F*(5,115) = 3.47, *p* = .043, η_p_^2^ = .13], which produced a marginally significant interaction with the consistency of the smile [*F*(5,115) = 2.67, *p* = .073, η_p_^2^ = .10] which is driven by increased activity predominantly from 2500ms onwards for consistent smiles, with a particular peak at 3000ms (*p* = .070).

#### Frowns

Corrugator: There were no significant differences in muscle activity when viewing consistent or inconsistent frowns [*F*(1,23) = 0.25, *p* = .63, η_p_^2^ = .011]. A main effect of Time was obtained [*F*(5,115) = 5.01, *p* = .007, η_p_^2^ = .18], and a marginal interaction of Consistency and Time [*F*(5,115) = 2.53, *p* = .067, η_p_^2^ = .099].

Zygomaticus: There were no significant differences in muscle activity when viewing consistent or inconsistent frowns [*F*(1,23) = 0.66, *p* = .43, η_p_^2^ = .028]. Although a main effect of Time was obtained [*F*(5,115) = 5.31, *p* = .012, η_p_^2^ = .19], there was no interaction of Consistency and Time [*F*(5,115) = 1.05, *p* = .35, η_p_^2^ = .044].

**Fig 8:**
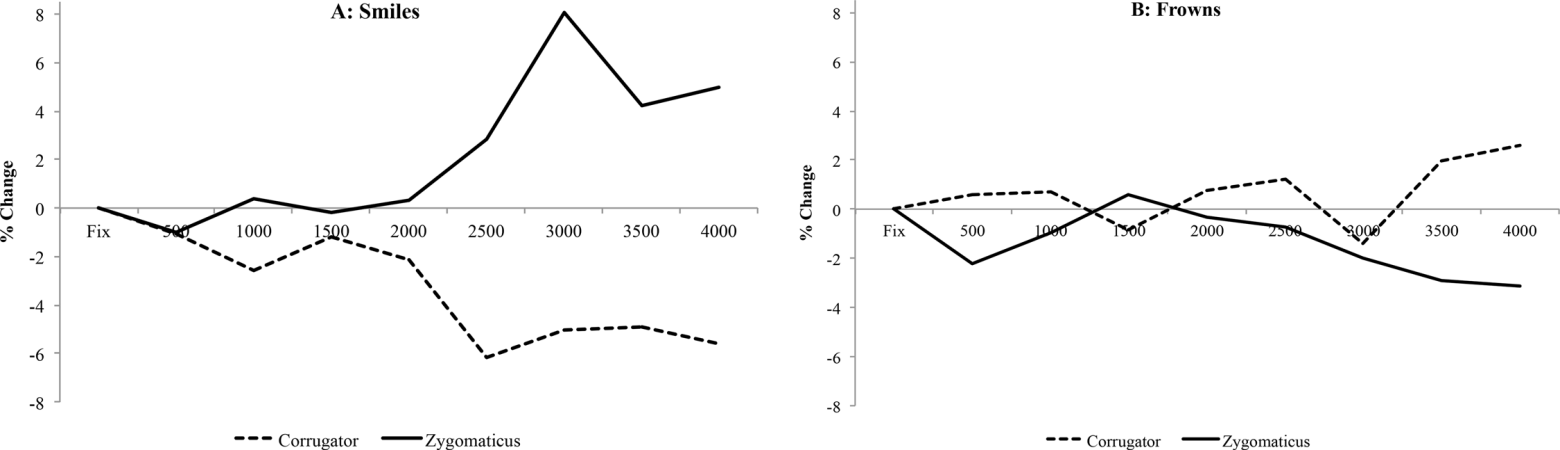
Analysis within emotion. Panel A shows analysis of smiles. This shows a difference score between consistent–inconsistent activity within each muscle to smiles. Panel B shows the same calculation, but between consistent–inconsistent frowns.

The within expression analysis appears to show that discrimination between consistent and inconsistent emotions is mostly detected when participants are observing smiles. That is, the corrugator is more active when viewing inconsistent than consistent smiles, whereas the zygomaticus trends in the opposite direction with greater activity to the consistent than inconsistent smiles (see Panel A in Fig 8). This pattern would reflect more negative emotions when viewing smiles that are inconsistent with the current context: that is, smiling at negative scenes. In contrast, this analysis provided less evidence for discrimination of consistent and inconsistent frowns.

In summary, a number of measures have been taken involving and relating to the implicit learning stage and these confirm that the face-identity consistency was encoded. That is, RTs to classify the scene are slower when the face emotion mismatches, causing response conflict; trust of inconsistent faces declines after learning; and mimicry of face emotion (EMG) is reduced when the face and scene emotion are inconsistent and this is especially the case when viewing smiling faces. We are now in a stronger position to see whether implicit encoding of face emotion consistency influences emotion mimicry when the face is later encountered with no contextual information, and therefore the participant is reliant upon their memory of each face and its emotional consistency. If the consistency does influence later emotion mimicry we expect to see an interaction between mimicry and consistent vs. inconsistent emotions, with the latter producing reduced mimicry.

### Retrieval Stage

Analyses obtained during this stage were performed in an identical process to that of the Learning Stage with an additional two time periods. Analyses were conducted using a repeated measures ANOVA with factors of Expression (smile and frown) and Consistency (Consistent and Inconsistent), with an additional factor of Time in EMG analyses.

#### Reaction times

All incorrect trials and those with RTs of < 200ms or >2500ms were removed prior to analysis. There was no main effect of Consistency with similar RTs for consistent (M = 1130ms, SE = 41.24) and inconsistent (M = 1139ms, SE = 38.21) trials [*F*(1,26) = 0.54, *p* = .47, η_p_^2^ = .020], nor Expression (frown: M = 1149ms, SE = 40.67; smile: M = 1120ms, SE = 40.33) [*F*(1,26) = 2.58, *p* = .12, η_p_^2^ = .090]. There was also no interaction between Consistency and Expression [*F*(1,26) = 0.11, *p* = .74, η_p_^2^ = .004]. Therefore the prior emotion-consistency of a particular individual does not affect the speed to categorise their emotion during a subsequent encounter.

#### Error rates

All Expression and Consistency pairings resulted in minimal erroneous trials with no one condition producing any greater average than 3%. There was no main effect of Consistency with similar errors for both consistent (M = 1.62%, SE = 0.56) and inconsistent (M = 2.14%, SE = 0.71) trials [*F*(1,26) = 0.60, *p* = .45, η_p_^2^ = .023], nor Expression (frown: M = 1.45%, SE = 0.57; smile: M = 2.31%, SE = 0.66) [*F*(1,26) = 2.34, *p* = .14, η_p_^2^ = .083]. There was also no interaction between Consistency and Expression [*F*(1,26) = 0.57, *p* = .46, η_p_^2^ = .021].

#### EMG–corrugator

**Fig 9:**
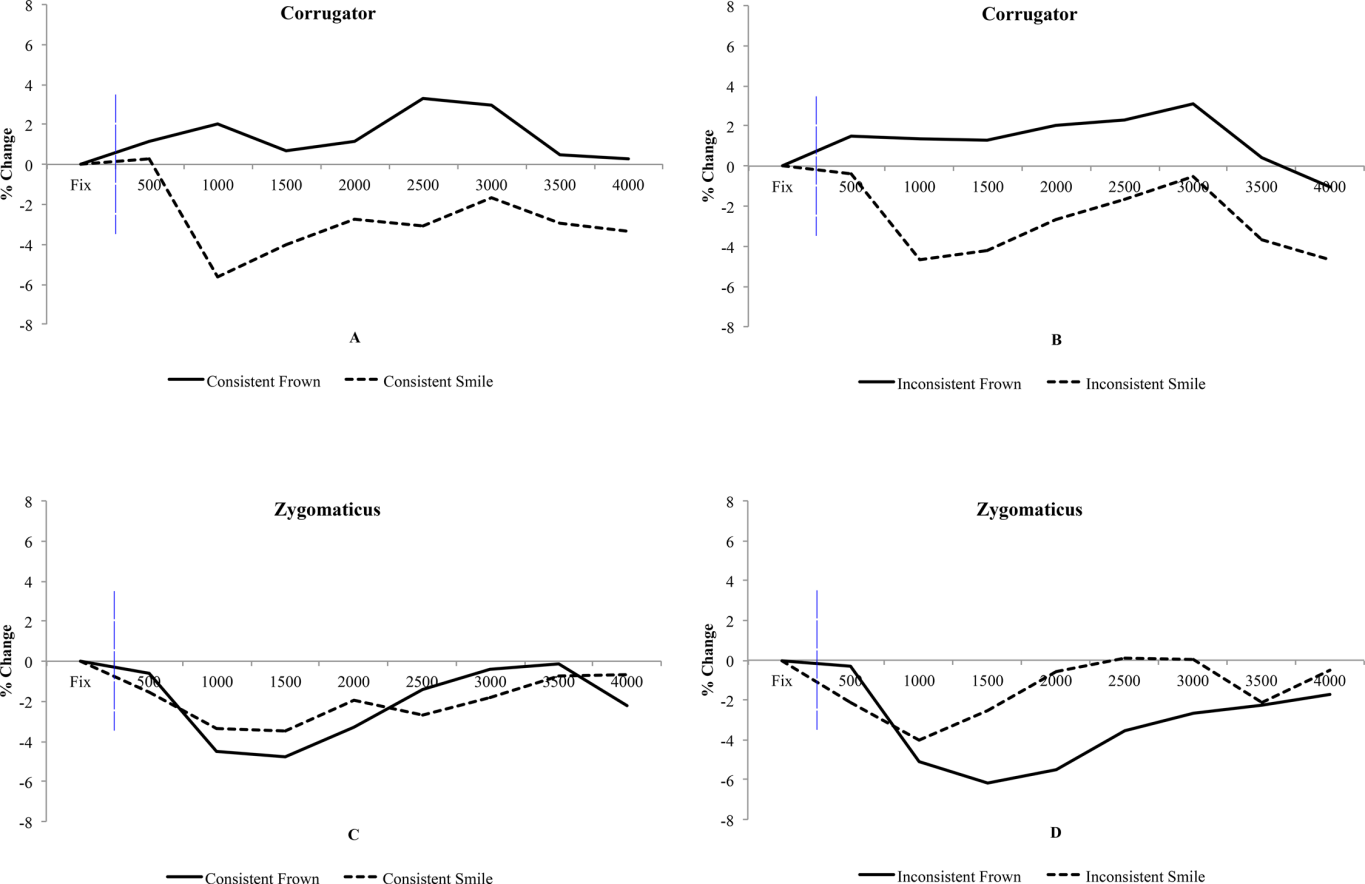
Retrieval Stage EMG. Time course graphs detailing muscle activity from the corrugator (A and B) and zygomaticus (C and D) toward Expressions and factors of Consistency. Blue vertical line illustrates the start point of facial expression morph.

As expected, when viewing a frowning face there was greater corrugator activity than when viewing a smiling face [*F*(1,23) = 19.10, *p* < .001, η_p_^2^ = .45]. There was no main effect of Consistency [*F*(1,23) = 0.001, *p* = .99, η_p_^2^< .001], although there was a marginal main effect of Time [*F*(7,161) = 2.42, *p* = .052, η_p_^2^ = .095]. Of central importance, there was no interaction of Expression and Consistency [F(1,23) = 0.014, p = .91, η_p_^2^ = .001], nor Expression x Consistency x Time [*F*(7,161) = 0.69, *p* = .56, η_p_^2^ = .029]. See Fig 9A and 9B for details.

#### EMG–zygomaticus

There was no main effect of Expression [*F*(1,23) = 0.96, *p* = .34, η_p_^2^ = .040], with similar relative muscle activity towards smiles and frowns. There was no main effect of Consistency [*F*(1,23) = 0.17, *p* = .69, η_p_^2^ = .007]. A main effect of Time was observed [*F*(7,161) = 3.51, *p* = .015, η_p_^2^ = .13]. Additionally, there was no significant interaction of Expression and Consistency [*F*(1,23) = 0.97, *p* = .33, η_p_^2^ = .041], nor Expression x Consistency x Time [*F*(7,161) = 1.62, *p* = .19, η_p_^2^ = 0.066]. See Fig 9C and 9D for details.

The results of the study are relatively clear. There is evidence that the consistency of the face emotion is computed during the implicit learning stage of the task, as differences between consistent and inconsistent emotions are observed in RTs, trust ratings and EMG. However, when we examine mimicry responses when these same faces are subsequently viewed with no contextual detail, there is no evidence that the prior emotional consistency of a particular face has any effect. Note that the duration between this subsequent viewing of the faces and the prior emotional consistency training is only a few minutes and during this time participants are not exposed to any new or novel information. Hence, even though an inconsistent face is rated as less trustworthy, for example, both the corrugator and the zygomaticus muscles reveal no evidence for an interaction between face emotion consistency and EMG mimicry.

## General Discussion

Emotional mimicry is a relatively fast process capable of forming cohesive social bonds and affiliations. Nevertheless it is still evident in situations where bonding is not a focus, or where the presentation of an emotion that is mimicked is shown subliminally. This therefore leads to the assertion that emotional mimicry, although undeniably beneficial in some situations, may be performed in an automatic and therefore unconscious manner. That is, a range of studies have demonstrated that emotional mimicry is a fast and spontaneous action [5] that can occur without conscious recognition, effort or intention [7]. In contrast, there are moderated automaticity accounts [4] whereby any mimicry is dependent upon contextual associations toward the emotion-expresser [13–16].

The present results are in line with accounts of moderated automaticity. That is, in the learning stage mimicry of another person’s emotion is automatic in that it is activated while ignoring a face. On the other hand, some features of the emotional environment in which the face is perceived do influence mimicry. That is, if the face emotion did not match the emotional properties of the target scene mimicry was suppressed. This contrasts with other work where context is manipulated in a more explicit manner. For example, in the current study each face was not directly specified as being a member of either an in-group or out-group, or of any competition or co-operation (factors which can impact mimicry: [15–16]). Rather, participants made these distinctions and assignments themselves based upon the incidental learning of the consistency of expressions and scene valence.

During the later retrieval stage, participants were tasked with categorising expressions shown by each of the faces with no additional scenes or consistency information. It was theorised that since participants had implicitly learnt and committed to memory the emotional consistency of the faces (as evidenced by reduced trustworthiness ratings) then mimicry would be reduced for faces that previously expressed inappropriate emotional responses. This was not the case. Regardless of the previous emotional consistency there were strong mimicry effects within the corrugator muscle to all faces, in that activity increased when viewing all frowns and decreased to all smiles.

This lack of retrieval effect probably reflects the form of learning. In this study it was implicit, where the faces were ignored and irrelevant to the main task of scene analysis. Note that in one of our unpublished studies we have also observed the sharp contrast between implicit and explicit learning of emotion consistency. That is, in contrast to the current data we observed powerful effects of explicit manipulations of the consistency of emotion. For example, when face emotion was appropriate, smiling at a person’s good news and frowning at their bad news, those individuals were mimicked. However, in a context where an interaction was negative, where a person smiled at another person’s bad news (schadenfreude) and frowned at another’s good news, mimicry was completely suppressed (see mimicry data EMG graphs in S1 Fig).

Finally we also note some intriguing asymmetries in our results. First, during the learning stage where faces expressed emotions that were either consistent or inconsistent with the emotion of a target scene, the contrasts were most salient when viewing smiling faces. That is, the zygomaticus shows marginally greater activity for an emotionally consistent smile than for an inconsistent smile, while corrugator showed the opposite pattern of greater activity for inconsistent than consistent smiles (See Panel A in Fig 8). This reflects the more negative representation of the inconsistent smiling face. However, when observing frowns, participant mimicry did not discriminate between consistent and inconsistent emotions. Second, during the retrieval stage there was no evidence for mimicry in the zygomaticus muscle. As just noted the zygomaticus muscle did discriminate between viewing smiles and frowns in the consistent emotion conditions of the training phase (Fig 7C) and the similar study reported in S1 Fig.

Hence the lack of effect during later face processing is not due to an insensitive measurement. Rather, we suspect that these two unusual results of only discriminating consistent from inconsistent emotions when viewing smiling faces during learning, and the lack of effect in the zygomaticus muscle during the retrieval stage, might be due to the general negative emotional context of the implicit learning task. That is, in typical social interactions face emotion is extremely reliable. When a smile is observed we can be sure that this is a response to some positive situation, whereas a frown almost always reflects a negative situation. Hence these emotional cues can always be relied on. However, in our current study participants can no longer rely on such social cues. Expressed emotions do not reflect the current situation on 50% of occasions. This is likely to have created a negative emotional context where other people can no longer be trusted.

This emotional mismatch clearly caused conflict during the implicit learning task as observed in the slowed RTs, reduced trust, and inhibited EMG responses during emotion mismatch trials. It is also noteworthy that even the consistent faces during the learning stage have a small decline in their level of trustworthiness. This is opposite to what might be expected from mere exposure effects, where repeated exposure to a stimulus increases positive emotions [32]. We hypothesise that this pervasive negative state prevented discrimination of the consistent-inconsistent frowning faces, but facilitated discrimination of the contrasting smiling faces; and during later retrieval the general negative emotional state inhibited response of the zygomaticus muscle associated with smiling. Certainly there is evidence that the zygomaticus is under more voluntary control than the corrugator [29–30], which does discriminate emotions during the retrieval stage of the experiment

## Conclusions

In sum, expressed emotions are typically extremely reliable: people’s smiles usually reflect positive environmental events and frowns reflect negative events. However, we demonstrate that in situations where emotions are not always reliable, even when a face is irrelevant and to-be-ignored, its emotion is computed in terms of its consistency with the current context. If a face expresses an inappropriate emotion, such as smiling at a negative scene, RTs to analyse the scene are slowed, there is reduced trust of people who consistently express inappropriate emotions, and facial mimicry of inappropriate emotions is suppressed. However, during subsequent presentation of these people who always expressed consistent or inconsistent emotions, mimicry is observed for both groups. Hence implicit incidental learning of a person’s reliability in expressing consistent emotions does not affect later processes, unlike effects with explicit manipulations of person properties. Finally, the surprising asymmetries in our data, such as the discrimination of the consistency of smiling faces but not frowning during learning, and the lack of the mimicry of the zygomaticus muscles associated with smiling during retrieval, may be caused by the general negative affect evoked by loss of trust in emotion reliability

## Supporting Information

S1 Table Trust rating stage data. Means, standard errors and standard deviations for ratings of trustworthiness according to face expression consistency and time of rating (either Pre, or Post Implicit learning stage).
(PDF)

S2 Table Implicit learning stage behavioural data. Means, standard errors and standard deviations for scene classification trials in the Implicit learning stage.
(PDF)

S3 Table Implicit learning stage EMG data. Means, standard errors and standard deviations for all expression types and time windows for corrugator and zygomaticus muscles.
(PDF)

S4 Table Retrieval stage behavioural data. Means, standard errors and standard deviations for expression classification trials in the Retrieval stage.
(PDF)

S5 Table Retrieval stage EMG data. Means, standard errors and standard deviations for all expression types and time windows for corrugator and zygomaticus muscles.
(PDF)

S1 Fig Additional experiment EMG activity. This figure shows mimicry effects when consistent and inconsistent emotions were manipulated in a more explicit manner. That is, participants were randomly assigned into two compatibility conditions. In the compatible condition each participant was told to imagine a situation where they had performed well in an exam. Their friends would be smiling at their success and their enemies would be frowning because they were unhappy at the participant doing well. In the incompatible condition this was reversed (smiles having negative connotations, and frowns having positive connotations). Participants were told to embody a situation where they had performed badly in an exam. Their friends would be equally disappointed and therefore would frown in support, whilst their enemies would be happy that the participant had performed badly and so would be smiling. By using this setup we have two distinct compatibility variations where emotional expressions reference their ‘traditional’ meaning in the compatible condition (smiles are good, frowns are bad) and where they reference their opposite meaning in the incompatible condition (frowns are good as supportive, smiles are bad as expressing pleasure/schadenfreude from misfortune). Note that this study had similar power to the implicit learning study, with 24 participants in each. Hence it highlights the contrast in the zygomaticus muscle sensitivity, which is significant in this explicit procedure, but shows no mimicry in the implicit retrieval procedure.
(TIF)
