# Supplementary material for: Facial Mimicry and Emotion Consistency: Influences of Memory and Context
Source: PLoS One. 2015 Dec 23;10(12):e0145731. doi: 10.1371/journal.pone.0145731 (PMC4689420; doi:10.1371/journal.pone.0145731)
Supplement: S1 Table — Means, standard errors and standard deviations for ratings of trustworthiness according to face expression consistency and time of rating (either Pre, or Post Implicit learning stage). (PDF) [file pone.0145731.s001.pdf]

S1 Table. Trust rating stage data. Means, standard errors and standard deviations for ratings of trustworthiness according to face expression consistency and time of rating (either Pre, or Post Implicit learning stage).

Time	Consistency	Mean	SE	SD
Pre	Consistent	-0.01	5.42	28.68
	Inconsistent	-0.84	4.83	25.57
Post	Consistent	-2.17	5.74	30.38
	Inconsistent	-13.75	5.01	26.52
