# Supplementary material for: Facial Mimicry and Emotion Consistency: Influences of Memory and Context
Source: PLoS One. 2015 Dec 23;10(12):e0145731. doi: 10.1371/journal.pone.0145731 (PMC4689420; doi:10.1371/journal.pone.0145731)
Supplement: S2 Table — Means, standard errors and standard deviations for scene classification trials in the Implicit learning stage. (PDF) [file pone.0145731.s002.pdf]

S2 Table. Implicit learning stage behavioural data. Means, standard errors and standard deviations for scene classification trials in the Implicit learning stage.

Measure	Consistency	Scene	Mean	SE	SD
RT (ms)	Consistent	Negative	919	41.08	213.46
		Positive	936	44.29	230.16
	Inconsistent	Negative	959	40.55	210.70
		Positive	1014	44.57	231.60
Error (%)	Consistent	Negative	1.85	0.66	3.44
		Positive	1.85	0.61	3.15
	Inconsistent	Negative	3.52	1.41	7.31
		Positive	4.44	1.29	6.70

Data is shown according to scene valence and expression consistency pairings. Reported measures include reaction time (RT) data measured in ms, and error response data reported as a % of trials.
