# Supplementary material for: Facial Mimicry and Emotion Consistency: Influences of Memory and Context
Source: PLoS One. 2015 Dec 23;10(12):e0145731. doi: 10.1371/journal.pone.0145731 (PMC4689420; doi:10.1371/journal.pone.0145731)
Supplement: S4 Table — Means, standard errors and standard deviations for expression classification trials in the Retrieval stage. (PDF) [file pone.0145731.s004.pdf]

S4 Table. Retrieval stage behavioural data. Means, standard errors and standard deviations for expression classification trials in the Retrieval stage.

Measure	Consistency	Expression	Mean	SE	SD
RT (ms)	Consistent	Frown	1143	42.98	223.34
		Smile	1118	43.21	224.51
	Inconsistent	Frown	1156	39.68	206.20
		Smile	1122	39.83	206.98
Error (%)	Consistent	Frown	1.39	0.61	3.16
		Smile	1.85	0.73	3.80
	Inconsistent	Frown	1.50	0.73	3.81
		Smile	2.78	0.90	4.69

Data is shown according to expression and prior scene-expression consistency pairings. Reported measures include reaction time (RT) data measured in ms, and error response data reported as a % of trials.
