# Supplementary material for: Facial Mimicry and Emotion Consistency: Influences of Memory and Context
Source: PLoS One. 2015 Dec 23;10(12):e0145731. doi: 10.1371/journal.pone.0145731 (PMC4689420; doi:10.1371/journal.pone.0145731)
Supplement: S5 Table — Means, standard errors and standard deviations for all expression types and time windows for corrugator and zygomaticus muscles. (PDF) [file pone.0145731.s005.pdf]

S5 Table. Retrieval stage EMG data. Means, standard errors and standard deviations for all expression types and time windows for corrugator and zygomaticus muscles.

				Time during trial (ms)								
Muscle	Consistency	Expression	Measure	Fixation	500	1000	1500	2000	2500	3000	3500	4000
Corrugator	Consistent	Smile	Mean	0.00	0.25	-5.64	-4.04	-2.77	-3.06	-1.67	-2.92	-3.33
			SE	0.00	0.89	1.83	1.85	1.69	1.54	1.39	1.53	1.59
			SD	0.00	4.38	8.97	9.08	8.29	7.54	6.81	7.51	7.78
		Frown	Mean	0.00	1.15	2.02	0.69	1.15	3.33	2.96	0.46	0.25
			SE	0.00	1.06	1.43	1.66	1.40	1.58	1.41	1.48	1.72
			SD	0.00	5.21	7.02	8.13	6.84	7.75	6.92	7.27	8.42
	Inconsistent	Smile	Mean	0.00	-0.38	-4.66	-4.18	-2.65	-1.64	-0.53	-3.66	-4.66
			SE	0.00	0.99	1.51	1.55	1.64	1.72	1.35	1.19	1.15
			SD	0.00	4.84	7.40	7.61	8.05	8.40	6.64	5.83	5.65
		Frown	Mean	0.00	1.52	1.37	1.26	2.05	2.27	3.12	0.42	-1.04
			SE	0.00	1.24	1.89	1.47	1.59	1.75	2.15	1.63	1.49
			SD	0.00	6.09	9.28	7.21	7.80	8.57	10.52	7.97	7.28
Zygomaticus	Consistent	Smile	Mean	0.00	-1.55	-3.35	-3.48	-1.95	-2.71	-1.82	-0.71	-0.68
			SE	0.00	1.25	1.45	1.23	1.68	1.73	1.86	1.76	1.61
			SD	0.00	6.11	7.11	6.02	8.22	8.48	9.09	8.62	7.90
		Frown	Mean	0.00	-0.63	-4.50	-4.79	-3.30	-1.39	-0.43	-0.11	-2.23
			SE	0.00	0.93	1.20	1.31	1.40	1.34	1.14	1.71	1.43
			SD	0.00	4.55	5.89	6.40	6.85	6.56	5.57	8.37	7.01
	Inconsistent	Smile	Mean	0.00	-2.10	-4.03	-2.52	-0.58	0.12	0.05	-2.12	-0.51
			SE	0.00	1.00	1.48	1.61	1.50	1.87	1.99	1.85	1.90
			SD	0.00	4.92	7.24	7.90	7.34	9.17	9.74	9.09	9.30
	Inconsistent	Frown	Mean	0.00	-0.29	-5.12	-6.17	-5.52	-3.53	-2.66	-2.23	-1.70
			SE	0.00	0.96	1.14	1.48	1.55	1.38	1.89	1.73	1.93
			SD	0.00	4.69	5.58	7.26	7.60	6.78	9.24	8.50	9.47
