# Supplementary figures and images for: Facial Mimicry and Emotion Consistency: Influences of Memory and Context
Source: PLoS One. 2015 Dec 23;10(12):e0145731. doi: 10.1371/journal.pone.0145731 (PMC4689420; doi:10.1371/journal.pone.0145731)

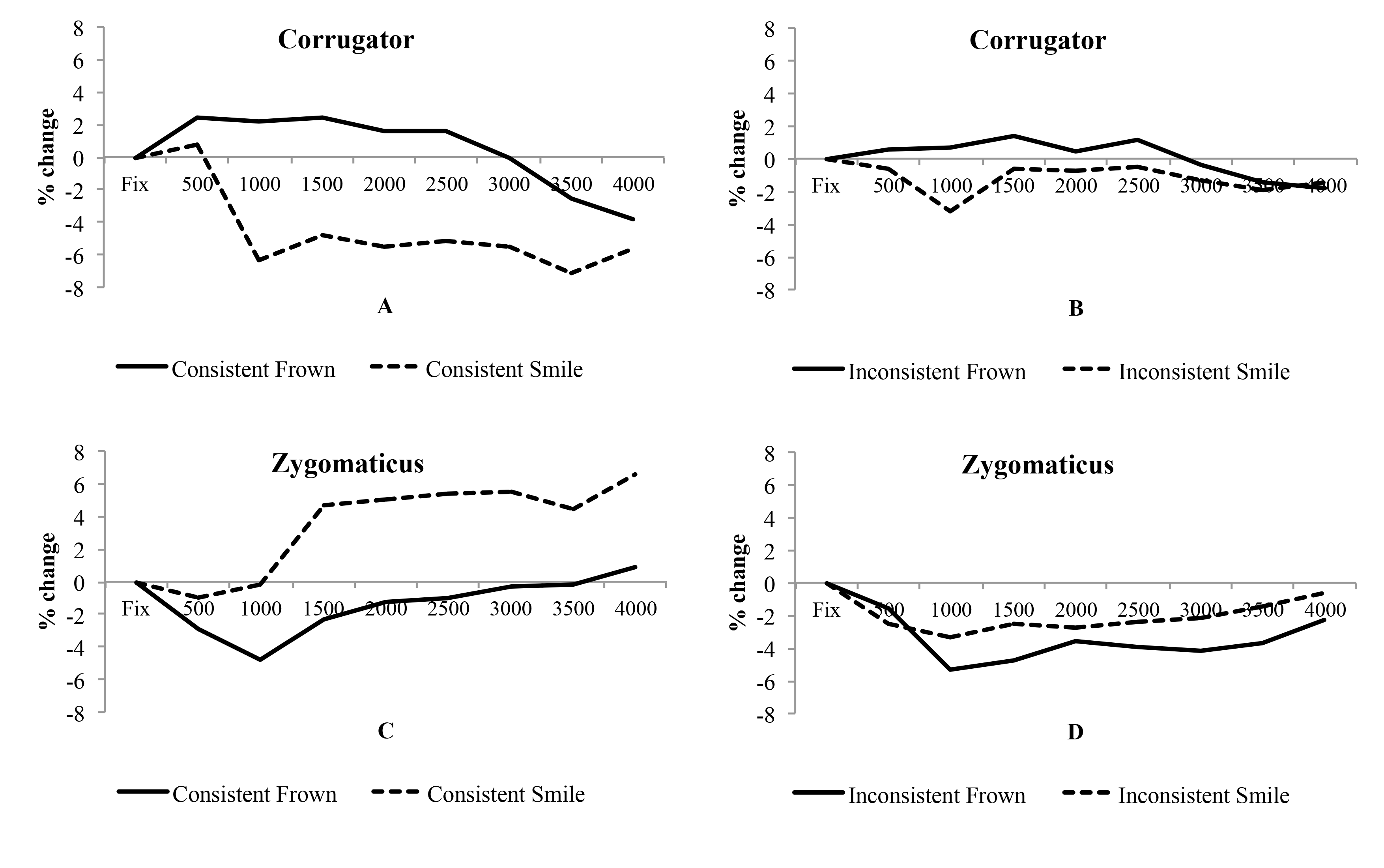

Supplement: S1 Fig — This figure shows mimicry effects when consistent and inconsistent emotions were manipulated in a more explicit manner. That is, participants were randomly assigned into two compatibility conditions. In the compatible condition each participant was told to imagine a situation where they had performed well in an exam. Their friends would be smiling at their success and their enemies would be frowning because they were unhappy at the participant doing well. In the incompatible condition this was reversed (smiles having negative connotations, and frowns having positive connotations). Participants were told to embody a situation where they had performed badly in an exam. Their friends would be equally disappointed and therefore would frown in support, whilst their enemies would be happy that the participant had performed badly and so would be smiling. By using this setup we have two distinct compatibility variations where emotional expressions reference their ‘traditional’ meaning in the compatible condition (smiles are good, frowns are bad) and where they reference their opposite meaning in the incompatible condition (frowns are good as supportive, smiles are bad as expressing pleasure/schadenfreude from misfortune). Note that this study had similar power to the implicit learning study, with 24 participants in each. Hence it highlights the contrast in the zygomaticus muscle sensitivity, which is significant in this explicit procedure, but shows no mimicry in the implicit retrieval procedure. (TIF) [file pone.0145731.s006.tif]
